# AI Digital Pathology Using qFibrosis Shows Heterogeneity of Fibrosis Regression in Patients with Chronic Hepatitis B and C with Viral Response

**DOI:** 10.3390/diagnostics14161837

**Published:** 2024-08-22

**Authors:** Feng Liu, Yameng Sun, Dean Tai, Yayun Ren, Elaine L. K. Chng, Aileen Wee, Pierre Bedossa, Rui Huang, Jian Wang, Lai Wei, Hong You, Huiying Rao

**Affiliations:** 1 Peking University People’s Hospital, Peking University Hepatology Institute, Infectious Disease and Hepatology Center of Peking University People’s Hospital, Beijing Key Laboratory of Hepatitis C and Immunotherapy for Liver Diseases, Beijing International Cooperation Base for Science and Technology on NAFLD Diagnosis, Beijing 100044, China; liufeng@pkuph.edu.cn (F.L.); strangehead@163.com (R.H.); wj.35@163.com (J.W.); 2 Liver Research Center, Beijing Friendship Hospital, Capital Medical University, 95 Yong-an Road, Xi-Cheng District, Beijing 100050, China; sunyamenggo@163.com; 3 HistoIndex Pte. Ltd., Singapore 117674, Singapore; dean.tai@histoindex.com (D.T.); elaine.chng@histoindex.com (E.L.K.C.); 4 Department of Pathology, National University Hospital, 5 Lower Kent Ridge Road, Singapore 119074, Singapore; 5 LiverPat SAS, F-75116 Paris, France; pierre.bedossa@liverpat.com; 6 Hepatopancreatobiliary Center, Beijing Tsinghua Changgung Hospital, Tsinghua University, Beijing 102218, China; weilai@mail.tsinghua.edu.cn

**Keywords:** artificial intelligence, chronic hepatitis B, chronic hepatitis C, liver fibrosis, qFibrosis, viral hepatitis

## Abstract

This study aimed to understand the dynamic changes in fibrosis and its relationship with the evaluation of post-treatment viral hepatitis using qFibrosis. A total of 158 paired pre- and post-treatment liver samples from patients with chronic hepatitis B (CHB; *n* = 100) and C (CHC; *n* = 58) were examined. qFibrosis was employed with artificial intelligence (AI) to analyze the fibrosis dynamics in the portal tract (PT), periportal (PP), midzonal, pericentral, and central vein (CV) regions. All patients with CHB achieved a virological response after 78 weeks of treatment, whereas patients with CHC achieved a sustained viral response after 24 weeks. For patients initially staged as F5/6 (Ishak system) at baseline, the post-treatment cases exhibited a significant reduction in the collagen proportionate area (CPA) (25–69%) and number of collagen strings (#string) (9–72%) across all regions. In contrast, those initially staged as F3/4 at baseline showed a similar CPA and #string trend at 24 weeks. For regression patients, 27 parameters (25 in the CV region) in patients staged as F3/4 and 15 parameters (three in the PT and 12 in the PP regions) in those staged as F5/6 showed significant differences between the CHB and CHC groups at baseline. Following successful antiviral treatment, the pre- and post-treatment liver samples provided quantitative evidence of the heterogeneity of fibrotic features. qFibrosis has the potential to provide new insights into the characteristics of fibrosis regression in both patients with CHB and CHC as early as 24 weeks after antiviral therapy.

## 1. Introduction

Liver fibrosis is characterized by the excessive accumulation of extracellular matrix proteins. Advanced liver fibrosis leads to cirrhosis and liver-related complications [1,2,3]. Therefore, there is an urgent need to develop more effective treatments to reverse liver fibrosis and prevent its progression to cirrhosis [4,5,6]. Furthermore, liver fibrosis evaluation is crucial for delineating fibrosis progression and predicting treatment efficacy [7,8].

Despite the utility of non-invasive techniques [9,10,11], including combined scores (e.g., Fibrosis Score 4), serum-based laboratory biomarkers (aspartate aminotransferase/Platelet Ratio Index), and radiological investigations (elastography techniques), limitations exist in evaluating mild to moderate fibrosis and in cases with progression or regression. Thus, liver biopsy remains the gold standard for evaluating liver fibrosis [12,13,14]. Hepatitis B and C viral infections are the leading causes of liver fibrosis. Advances in effective antiviral therapies have led to several studies [15,16] reporting decreases in fibrosis and, in some cases, complete resolution, even among patients with cirrhosis. However, in these patients, liver fibrosis is an evolving process, and the assessment of liver biopsy may reveal complex fibrosis patterns involving both progressive and regressive features. Currently, classification systems, such as Ishak and METAVIR systems, have been developed as discrete, semiquantitative staging tools for fibrosis employing a stepwise discrimination analysis. However, these systems lack the granularity required to examine the heterogeneity of fibrosis features and are also affected by intra- and inter-observer variability [17,18]. Therefore, a technology that can quantitatively and dynamically evaluate fibrosis should be at the core of fibrosis evaluation, thus enhancing the value and importance of liver biopsy.

Recent developments in digital pathology and full-slide imaging, including second harmonic generation (SHG)/two-photon excitation fluorescence (TPEF) microscopy, offer refined insights into collagen dynamics within liver tissues [19,20,21,22]. qFibrosis is an artificial intelligence (AI)-based digital pathology approach which leverages AI to quantitatively analyze fibrosis on a continuous scale, providing a detailed assessment of collagen characteristics including morphology (length and width) and location (portal vein and central vein). This technology enhances the utility of liver biopsies by enabling a detailed analysis beyond traditional scoring systems, thereby offering a deeper understanding of the fibrosis patterns associated with both disease progression and regression in indications such as chronic hepatitis B (CHB) and non-alcoholic fatty liver disease (NAFLD) [21,22,23,24].

This study aims to explore the dynamic changes in liver fibrosis post-treatment in patients with viral hepatitis using the qFibrosis method, focusing on detailed collagen morphology and distribution to better understand the heterogeneity of fibrosis changes, which are crucial for precise disease management.

## 2. Materials and Methods

### 2.1. Study Population

A total of 158 consecutive series of paired pre- and post-treatment liver biopsies from patients with CHB (*n* = 100) and chronic hepatitis C (CHC; *n* = 58) were collected and examined. For the CHB cohort, liver samples were performed at both baseline and week 78 after Entecavir antiviral treatment; however, for the CHC cohort, liver samples were obtained at baseline and 24 weeks after direct-acting antiviral-based treatment. A virological response is defined as a compliant patient with HBV DNA levels below 2000 IU/mL after 78 weeks of treatment. Sustained virological response was defined as the non-detection of serum hepatitis C virus RNA levels after 24 weeks of treatment. Patients with other chronic liver diseases (such as alcohol-related liver disease, severe non-alcoholic fatty liver disease, autoimmune liver disease, and drug-induced liver injury) or incomplete clinical or laboratory data were excluded. Information on patient demographics, clinical results, laboratory results, and histological data were collected from medical records.

The research plan was approved by the Ethics Committee of Peking University People’s Hospital and Beijing Friendship Hospital (2020PHB039-01) and was conducted in accordance with the Helsinki Declaration. Written informed consent was obtained from all participants prior to study initiation.

### 2.2. Histological Assessments

All available 4 μm thick formalin-fixed, paraffin-embedded pre- and post-treatment histologic samples were routinely stained with hematoxylin and eosin, Masson trichrome, and reticulin stain. All slides were independently staged from 0 to 6 using the Ishak scoring system by experienced histopathologists [25]. The minimum length of all biopsy samples was 10 mm, with at least five portal veins.

### 2.3. Image Acquisition and Quantification

A total of 316 unstained liver samples were imaged using the Genesis system (HistoIndex Pte. Ltd., Singapore), which is a laser-based multiphoton fluorescence imaging microscope, where the SHG microscopy serves to visualize collagen and TPEF microscopy highlights hepatocytes. The details of the procedure have been described previously [22]. In brief, the samples were laser excited at 780 nm, TPEF signals were recorded at 550 nm, and SHG signals were recorded at 390 nm. Images were acquired at 20× magnification with resolutions of 512 × 512 pixels, and each image presented a 200 × 200 μm^2^ area of tissue. Multiple adjacent images were captured to cover the entire section.

The SHG/TPEF images were analyzed using a digital image processing algorithm, which is an AI-based algorithm that can detect collagen features in various regions of the liver tissue. The liver tissue region was detected from the TPEF channel, and tissue fragments with an area of less than 0.5 mm^2^ were removed from the analysis. Collagen was detected from the SHG channel using Otsu’s automatic threshold [26]. Portal tract (PT) and central vein (CV) regions were determined by a decision tree constructed using the Classification and Regression Tree (CART) [27]. The periportal (PP) region and the pericentral (PC) region are defined as being within 100 µm around the portal tract and central vein, respectively, and the region in between the PP and PC regions is the midzonal (MZ) region. Additionally, 184 fibrosis parameters such as the collagen proportionate area (CPA), number of collagen strings (#string), length of collagen strings (LengthStr), and width of collagen strings (WidthStr) were quantified in each region [28]. For the 200 CHB biopsies, the AI-based measurement showed an average length of 15.5 ± 5.4 mm, with an average number of portal tracts of 13.7 ± 8.8. And for the 116 CHC biopsies, the average length and number of portal tracts were 24.5 ± 9.8 mm and 17.8 ± 11.5, respectively. The liver specimens were well above the minimum sampling size required for qFibrosis analyses [29].

### 2.4. Analysis of Treated vs. Untreated Samples

Two types of comparisons were performed to assess the heterogeneity of fibrosis progression or regression. First, a paired analysis was conducted to calculate the relative changes in the fibrosis parameters at various regions before and after treatment for three cohorts of patients based on their Ishak scores at baseline: the early fibrosis cohort (F0/1/2), moderate fibrosis cohort (F3/4), and advanced fibrosis cohort (F5/6). The percentage of regressed patients was analyzed based on the fibrosis parameters in each region. Regression of fibrosis was defined as a decrease of ≥1 standard error of the mean from pre- to post-treatment [23]. Second, an unpaired analysis was conducted to calculate the relative difference in fibrosis parameters at various regions between treated and untreated samples within the same fibrosis stage (F0/1/2, F3/4, and F5/6).

### 2.5. Statistical Analysis

Statistical differences in fibrosis parameters (1) at various regions before and after treatment in the paired analysis and (2) at various regions between the treated and untreated samples within the same fibrosis stage from the unpaired analysis was assessed using the Wilcoxon signed-rank test. The proportion of patients showing fibrosis improvement in the collagen parameters at the five regions was analyzed. Fibrosis improvement was determined by examining the relative changes in collagen parameters that were reduced by more than 10% after treatment. The difference in the proportion of patients with fibrosis regression between the CHB and CHC groups was evaluated using the chi-square test. The statistical significance threshold was set to *p* < 0.05 [26].

## 3. Results

### 3.1. Demographic, Clinical, and Laboratory Characteristics of Patients

A total of 158 patients with paired liver biopsies were enrolled in this study. qFibrosis of paired liver biopsy samples from patients with CHB and CHC was carried out based on the AI measurement (Figure S1). The clinical and histological characteristics of all paired patients are summarized in Tables S1 and S2. In the CHB group, the mean age of patients was 37.8 ± 9.7 years, with males accounting for 78% (*n* = 78). Among them, 69% of the pre-treatment patients had Ishak scores ≥ 3; this decreased to 59% after treatment. In the CHC group, the mean age of patients was 41.5 ± 14.1 years, with males accounting for 36% (*n* = 21). Among them, 83% (*n* = 48) of pre-treatment patients had Ishak scores ≥ 3; this decreased to 71% (*n* = 41) after treatment. After antiviral therapy, significant declines in alanine aminotransferase, aspartate aminotransferase, and viral load were observed (*p* < 0.001). All patients with hepatitis B infection achieved a virological response after 78 weeks of treatment, with 69% of cases achieving a complete virological response (HBV DNA < 20 IU/mL). All patients with CHC achieved a sustained virological response after 24 weeks.

The fibrosis stage improved in 42% of patients with CHB, stabilized in 51%, and worsened in 7% after treatment compared with that before treatment. Similarly, the fibrosis stage improved in 40% of patients with CHC, stabilized in 60%, and worsened in 15%. Most of the observed differences were a one-stage change, with 80% exhibiting a single-stage improvement and 20% showing two- or three-stage improvements in the biopsy pairs (Figure S2).

### 3.2. Parameters between Pre- and Post-Treatment Liver Biopsies from Patients with Hepatitis B and C

**Figure 1:**
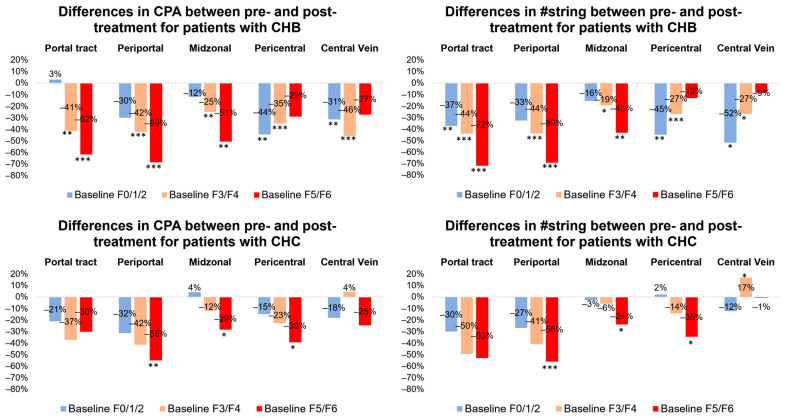
A paired analysis of the differences in the collagen proportionate area (CPA) and number of collagen strings (#string) between the pre- and post-treatment liver biopsies from patients with chronic hepatitis B and C. * *p* < 0.05, ** *p* < 0.01, and *** *p* < 0.001.

When comparing the collagen contents between the pre- and post-treatment liver biopsies, the post-treatment liver biopsies exhibited a significantly reduced area of collagen (Figure S3). In the samples staged as F5/6 at baseline, all post-treatment cases showed a significantly decreased CPA (25–69%) and #string (9–72%) across all regions, especially in the PT and PP regions. This result indicates a significant reduction in collagen associated with septa. For samples staged as F3/4 at baseline, a similar CPA and #string trend was observed, except for more CPA (4%) and #string (17%) in CV areas in the post-treatment cases with CHC at 24 weeks. This suggests that the duration was likely insufficient for the resolution of some portal-central bridges. In the F0/1/2 cohort, less CPA (12–44%) and #string (3–52%) were also observed across all regions, especially in the PC and CV regions in cases with CHB and in the PP and PC regions in cases with CHC. After 78 weeks of treatment, patients with CHB showed a 3% increase in CPA in the PT region (Figure 1). Similarly, after 24 weeks of treatment, patients with CHC exhibited a 4% increase in CPA in the MZ region and a 2% increase in #string in the PC region, indicating fibrous expansion in the perisinusoidal and PC areas.

For the unpaired analysis at the same fibrosis stage, CPA and #string mostly had a similar decreasing trend in different regions compared to the samples obtained post-treatment (Figure S4). Especially in the PP region, the post-treatment samples staged as F5/6 showed a significant lower CPA (47–70%) and #string (49–69%) than the pre-treatment samples with the same stages. The differences in fibrosis parameters for the same fibrosis stages between the treated and untreated samples reflected the heterogeneity of collagen changes in different regions of liver tissue after treatment, which cannot be reflected by conventional scoring systems.

**Figure 2:**
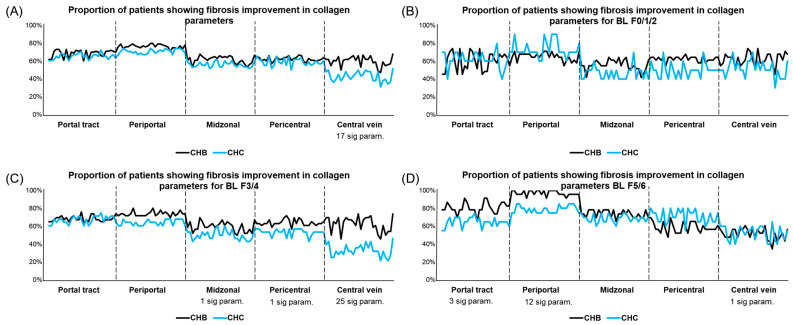
Proportion of patients showing fibrosis improvement in collagen parameters at five regions, including portal tract, periportal, midzonal, pericentral, and central vein regions. There were 28 parameters in each region, and 140 collagen parameters were analyzed. Fibrosis improvement was determined by examining relative changes in collagen parameters that were reduced by more than 10% after treatment. (**A**) Analysis was performed for all patients. (**B**–**D**) Analysis was performed for patients with baseline fibrosis stages of F0/1/2, F3/4, and F5/6, respectively. Number of parameters with significant differences (calculated using chi-square test) between patients with CHB and CHC was indicated. Statistical significance was set at *p* < 0.05.

Furthermore, all parameters in the PT, PP, MZ, PC, and CV regions were analyzed between patients with regressed CHB and CHC. Seventeen parameters (all in the CV regions) showed significant differences. The CHB group had a higher proportion of patients with regression than the CHC group. For samples staged as F3/4 at baseline, 27 parameters (25 in the CV regions) showed significant differences. For those staged as F5/6 at baseline, 15 parameters (three at the PT and 12 at the PP) showed significant differences (Figure 2 and Table S3).

## 4. Discussion

Assessing dynamic changes in liver fibrosis is crucial for distinguishing fibrosis progression, predicting the efficacy of liver fibrosis treatment, determining patient prognosis, and developing follow-up monitoring strategies. CHB and CHC viral infections are common causes of liver cirrhosis and associated hepatocellular carcinoma. In our study, the quantitative AI-based qFibrosis approach has the potential to provide new insights into monitoring dynamic changes in liver fibrosis in viral hepatitis cases as early as 24 weeks post-treatment.

Liver histology is considered the gold standard for staging fibrosis. In patients with CHB, treatment with a nucleotide analog or tenofovir disoproxil fumarate [30,31,32] can achieve fibrosis and cirrhosis regression according to Ishak’s scoring evaluation criteria. Similarly, CHC cases treated with interferon or direct-acting antiviral agents [33,34,35] can achieve fibrosis and cirrhosis regression, as assessed through METAVIR or Ishak staging. However, the two scoring systems are semiquantitative staging systems and do not measure changes in collagen architecture, correctly score fibrosis regression, or further capture subtle changes in fibrosis after treatment. The CPA is derived from calculating the percentage of collagen areas stained with Picro Sirius red over the total liver tissue. One of the main advantages of morphometrics is that it provides a definable quantitative scale, which is more accurate than semiquantitative scoring methods. Previous studies have shown changes in hepatic fibrosis after α-interferon therapy in patients with CHB and CHC by using morphometry [36]. In all of these studies, the decrease or increase in collagen was estimated pre- and post-treatment. However, the CPA measurement can be affected by technical issues, such as differences in staining procedures, operator experience, and imaging software used, all of which may interfere with image analysis [19,37].

The AI-based qFibrosis algorithm analyzed SHG/TPEF images from unstained slides, delivering a quantitative assessment of fibrotic changes on a continuous scale. This approach offers a more nuanced reflection of subtle variations compared to traditional semiquantitative scores. qFibrosis was previously established by Xu et al. [21] and incorporated spatial structural features related to pathology staging. Sun et al. [20] also demonstrated that qFibrosis could reveal otherwise undetected changes in collagen, allowing for the further subcategorization of collagen. In our study, consistent reductions were observed in collagen features at various spatial locations in patients with CHB and CHC after 78- or 24-week treatment regimens. This suggests that collagen resolved even after a short period of effective antiviral treatment. This is consistent with the results of Sun et al., who examined patients with CHB and showed that collagen reduction was obvious in the CV region of samples staged as F3/4 at baseline and in the portal region in those staged as F5/6 at baseline. This effect was more obvious with a prolonged period of effective antiviral therapy. This may be related to the long period of effective antiviral treatment for CHB and the different pathological mechanisms of fibrosis regression in patients with CHB and CHC. Greater portal and periportal fibrosis seen in patients with CHC [38] might be related to the insufficient resolution of portal-central bridging and portal fibrosis within a short time after treatment.

In stages F3/4 and F1/2, collagen features showed minor inconsistencies in spatial locations, such as an increase in #string in CV areas in F3/4 and the PP areas in F1/2 after 24 weeks of treatment. However, after a further analysis of the proportion of patients with regression, the results show reduced consistency in spatial locations, particularly in the CV and portal regions. This suggests that minor collagen changes should be differentiated and analyzed in patients with regression or progression. qFibrosis could provide more refined insights into collagen changes according to different causes of chronic liver disease, proving more sensitive and meaningful results when monitoring the dynamic fibrotic process of disease progression.

This study has some limitations. First, liver biopsies were examined mainly in Asian populations, and a larger cohort could provide more statistical power and generalizability of the findings. In the future, we plan to collect more samples from different ethnic groups to analyze patterns of qFibrosis changes. Second, based on the differences in collagen changes between patients with CHB and CHC, we aim to refine the incorporation of etiology-related features. Third, compared to traditional semiquantitative scoring systems and morphometry, feature recognition and quantification are expensive and time-consuming, requiring technical improvements that must be evaluated quickly. Fourth, fibrosis changes might have a certain correlation with clinical outcomes (i.e., liver cirrhosis and hepatocellular carcinoma), and the cutoff system of qFibrosis must be defined and characterized to determine whether the collagen changes are associated with improved clinical outcomes before the system is widely adopted.

## 5. Conclusions

In this study, we employed AI-based qFibrosis to better detect changes in collagen in CHB and CHC cohorts with chronic liver disease. The qFibrosis approach has the potential to provide new insights into the dynamic’s heterogeneity of fibrosis regression in patients with viral hepatitis as early as 24 weeks after antiviral therapy. Although much work remains to be conducted before routine clinical practice, the advances of qFibrosis in AI image analysis show great promise in quantifying and calculating different variables of collagen distribution, morphology, and structure. This algorithm can monitor the response to treatment and be used as a network-assisted evaluation tool to facilitate the further independent exploration of its clinical applicability for liver diseases, including not only CHB and CHC, but also fatty liver and other liver diseases.

## Data Availability

The deidentified participant data will be made available from the corresponding author (H.R.) upon reasonable request.

## Supplementary Materials

The following supporting information can be downloaded at https://www.mdpi.com/article/10.3390/diagnostics14161837/s1, Table S1: Clinical and histological characteristics of chronic hepatitis B patients before and after treatment (*n* = 100). Wilcoxon signed rank test was used; Table S2: Clinical and histological characteristics of chronic hepatitis C patients before and after treatment (*n* = 58); Table S3: The significant parameters for the analysis of regressive patients between CHB and CHC. The parameters with *p* < 0.05 were highlighted. Chi-square test was used for the analysis; Figure S1: Flowchart of qFibrosis based on AI analysis of paired liver biopsy samples from patients with CHB and CHC; Figure S2: Distribution of patients with fibrosis progression, stability and regression as determined by Ishak fibrosis score before and after treatment. CHB patients (A) and CHC patients (B). Fibrosis progression (yellow) or regression (green) was defined as increase or decrease in Ishak fibrosis score; fibrosis stability (brown) was defined as no change in Ishak fibrosis score compared with BL; Figure S3: Examples of pre- and post-treatment SHG/TPEF images from one CHB patient and one CHC patient. Both CHB and CHC patients had fibrosis improvement with 1-point Ishake stage and fibrosis regression at portal tract region (white arrow). CPA-PT, collagen proportionate area at portal tract region quantified by SHG/TPEF; Figure S4: Unpaired analysis—difference in collagen proportionate areas (CPA) and #string between treated and untreated liver biopsies from hepatitis B and C patients.

## Abbreviations

SHG/TPEF	second harmonic generation/two-photon excitation fluorescence
AI	artificial intelligence
CHB	chronic hepatitis B
CHC	chronic hepatitis C
PT	portal tract
PP	periportal
MZ	midzonal
PC	pericentral
CV	central vein
CPA	collagen proportionate area
#string	number of collagen strings

## References

[1] Friedman S.L. (2024). Hepatic Fibrosis and Cancer: The Silent Threats of Metabolic Syndrome. Diabetes Metab. J..

[2] Caligiuri A., Gentilini A., Pastore M., Gitto S., Marra F. (2021). Cellular and Molecular Mechanisms Underlying Liver Fibrosis Regression. Cells.

[3] Hammerich L., Tacke F. (2023). Hepatic inflammatory responses in liver fibrosis. Nat. Rev. Gastroenterol. Hepatol..

[4] Lemoinne S., Friedman S.L. (2019). New and emerging anti-fibrotic therapeutics entering or already in clinical trials in chronic liver diseases. Curr. Opin. Pharmacol..

[5] Roehlen N., Crouchet E., Baumert T.F. (2020). Liver fibrosis: Mechanistic concepts and therapeutic perspectives. Cells.

[6] Akkız H., Gieseler R.K., Canbay A. (2024). Liver Fibrosis: From Basic Science towards Clinical Progress, Focusing on the Central Role of Hepatic Stellate Cells. Int. J. Mol. Sci..

[7] Wang Y., Hou J.L. (2016). Fibrosis assessment: Impact on current management of chronic liver disease and application of quantitative invasive tools. Hepatol. Int..

[8] Henderson N.C., Rieder F., Wynn T.A. (2020). Fibrosis: From mechanisms to medicines. Nature.

[9] Loomba R., Adams L.A. (2020). Advances in non-invasive assessment of hepatic fibrosis. Gut.

[10] Anstee Q.M., Castera L., Loomba R. (2022). Impact of non-invasive biomarkers on hepatology practice: Past, present and future. J. Hepatol..

[11] Oeda S., Tanaka K., Oshima A., Matsumoto Y., Sueoka E., Takahashi H. (2020). Diagnostic Accuracy of FibroScan and Factors Affecting Measurements. Diagnostics.

[12] Bedossa P. (2018). Diagnosis of non-alcoholic fatty liver disease/non-alcoholic steatohepatitis: Why liver biopsy is essential. Liver Int..

[13] Chowdhury A.B., Mehta K.J. (2023). Liver biopsy for assessment of chronic liver diseases: A synopsis. Clin. Exp. Med..

[14] Torbenson M., Washington K. (2020). Pathology of liver disease: Advances in the last 50 years. Hum. Pathol..

[15] Block T.M., Chang K.M., Guo J.T. (2021). Prospects for the global elimination of hepatitis B. Annu. Rev. Virol..

[16] Rockey D.C., Friedman S.L. (2021). Fibrosis regression after eradication of hepatitis C virus: From bench to bedside. Gastroenterology.

[17] Fukusato T., Fukushima J., Shiga J., Takahashi Y., Nakano T., Maeyama S., Masayuki U., Ohbu M., Matsumoto T., Matsumoto K. (2005). Interobserver variation in the histopathological assessment of nonalcoholic steatohepatitis. Hepatol. Res..

[18] Davison B.A., Harrison S.A., Cotter G., Alkhouri N., Sanyal A., Edwards C., Colca J.R., Iwashita J., Koch G.G., Dittrich H.C. (2020). Suboptimal reliability of liver biopsy evaluation has implications for randomized clinical trials. J. Hepatol..

[19] Astbury S., Grove J.I., Dorward D.A., Guha I.N., Fallowfield J.A., Kendall T.J. (2021). Reliable computational quantification of liver fibrosis is compromised by inherent staining variation. J. Pathol. Clin. Res..

[20] Sun Y., Zhou J., Wu X., Chen Y., Piao H., Lu L., Ding H., Nan Y., Jiang W., Wang T. (2018). Quantitative assessment of liver fibrosis (qFibrosis) reveals precise outcomes in Ishak “stable” patients on anti-HBV therapy. Sci. Rep..

[21] Xu S., Wang Y., Tai D.C.S., Wang S., Cheng C.L., Peng Q., Yan J., Chen Y., Sun J., Liang X. (2014). qFibrosis: A fully-quantitative innovative method incorporating histological features to facilitate accurate fibrosis scoring in animal model and chronic hepatitis B patients. J. Hepatol..

[22] Liu F., Goh G.B., Tiniakos D., Wee A., Leow W.Q., Zhao J.M., Rao H.Y., Wang X.X., Wang Q., Wan W.K. (2020). qFIBS: An automated technique for quantitative evaluation of fibrosis, inflammation, ballooning, and steatosis in patients with nonalcoholic steatohepatitis. Hepatology.

[23] Naoumov N.V., Brees D., Loeffler J., Chng E., Ren Y., Lopez P., Tai D., Lamle S., Sanyal A.J. (2022). Digital pathology with artificial intelligence analyses provides greater insights into treatment-induced fibrosis regression in NASH. J. Hepatol..

[24] Sanyal A.J., Jha P., Kleiner D.E. (2024). Digital pathology for nonalcoholic steatohepatitis assessment. Nat. Rev. Gastroenterol. Hepatol..

[25] Ishak K., Baptista A., Bianchi L., Callea F., De Groote J., Gudat F., Denk H., Desmet V., Korb G., MacSween R.N. (1995). Histological grading and staging of chronic hepatitis. J. Hepatol..

[26] Otsu N. (1979). A Threshold selection method from gray-level histograms. IEEE Trans. Syst. Man Cybern. B Cybern..

[27] Lewis R.J. An Introduction to Classification and Regression Tree. (CART) Analysis. Proceedings of the Annual Meeting of the Society for Academic Emergency Medicine.

[28] Ng N., Tai D., Ren Y., Chng E., Seneshaw M., Mirshahi F., Idowu M., Asgharpour A., Sanyal A.J. (2023). Second-harmonic generated quantifiable fibrosis parameters provide signatures for disease progression and regression in nonalcoholic fatty liver disease. Clin. Pathol..

[29] Wang B., Sun Y., Zhou J., Wu X., Chen S., Shi Y., Wu S., Liu H., Ren Y., Ou X. (2019). SHG/TPEF-based image technology improves liver fibrosis assessment of minimally sized needle biopsies. Hepatol. Int..

[30] Chang T.T., Liaw Y.F., Wu S.S., Schiff E., Han K.H., Lai C.L., Safadi R., Lee S.S., Halota W., Goodman Z. (2010). Long-term entecavir therapy results in the reversal of fibrosis/cirrhosis and continued histological improvement in patients with chronic hepatitis B. Hepatology.

[31] Marcellin P., Gane E., Buti M., Afdhal N., Sievert W., Jacobson I.M., Washington M.K., Germanidis G., Flaherty J.F., Aguilar Schall R. (2013). Regression of cirrhosis during treatment with tenofovir disoproxil fumarate for chronic hepatitis B: A 5-year open-label follow-up study. Lancet.

[32] Isgro G., Calvaruso V., Andreana L., Luong T.V., Garcovich M., Manousou P., Alibrandi A., Maimone S., Marelli L., Davies N. (2013). The relationship between transient elastography and histological collagen proportionate area for assessing fibrosis in chronic viral hepatitis. J. Gastroenterol..

[33] Poynard T., McHutchison J., Manns M., Trepo C., Lindsay K., Goodman Z., Ling M.H., Albrecht J. (2002). Impact of pegylated interferon alfa-2b and ribavirin on liver fibrosis in patients with chronic hepatitis C. Gastroenterology.

[34] Martini S., Sacco M., Strona S., Arese D., Tandoi F., Dell Olio D., Stradella D., Cocchis D., Mirabella S., Rizza G. (2017). Impact of viral eradication with sofosbuvir-based therapy on the outcome of post-transplant hepatitis C with severe fibrosis. Liver Int..

[35] Mauro E., Crespo G., Montironi C., Londoño M.C., Hernández-Gea V., Ruiz P., Sastre L., Lombardo J., Mariño Z., Díaz A. (2018). Portal pressure and liver stiffness measurements in the prediction of fibrosis regression after sustained virological response in recurrent hepatitis C. Hepatology.

[36] D’Ambrosio R., Aghemo A., Rumi M.G., Ronchi G., Donato M.F., Paradis V., Colombo M., Bedossa P. (2012). A morphometric and immunohistochemical study to assess the benefit of a sustained virological response in hepatitis C virus patients with cirrhosis. Hepatology.

[37] Soon G., Wee A. (2021). Updates in the quantitative assessment of liver fibrosis for nonalcoholic fatty liver disease: Histological perspective. Clin. Mol. Hepatol..

[38] Zaitoun A.M., Al Mardini H., Awad S., Ukabam S., Makadisi S., Record C.O. (2001). Quantitative assessment of fibrosis and steatosis in liver biopsies from patients with chronic hepatitis C. J. Clin. Pathol..

