# Supplementary material for: AI Digital Pathology Using qFibrosis Shows Heterogeneity of Fibrosis Regression in Patients with Chronic Hepatitis B and C with Viral Response
Source: Diagnostics (Basel). 2024 Aug 22;14(16):1837. doi: 10.3390/diagnostics14161837 (PMC11353864; doi:10.3390/diagnostics14161837)

Supplementary material

Supplementary Table 1. Clinical and histological characteristics of chronic hepatitis B patients before and after treatment (n=100). Wilcoxon signed rank test was used

**Characteristics**	**Baseline n = 100 patients**	**Week 78 n = 100 patients**	***P* value**
Age(yr)	37.8±9.7	−	-
Male, n(%)	78(78%)	−	-
ALT, U/L	125.4±135.6	30.7±16.0	<0.001
AST, U/L	77.4±65.9	26.9±9.7	<0.001
Albumin, g/l	43±5.8	45.5±3.5	<0.001
TBIL(μmol/L)	17.8±13.9	13.7±5.6	<0.001
Platelets(×10^3μL)	169.8±55.6	163.7±62.4	0.586
INR, ratio	1.1±0.1	1.0±0.1	<0.001
HBV-DNA, Log IU/mL	6.4±1.6	0.5±0.8	<0.001
Ishak fibrosis stage, n (%)			<0.001
0	1	2	
1	8	11	
2	22	28	
3	23	29	
4	23	14	
5	19	15	
6	4	1	
HAI score, n (%)			<0.001
0–3	12	42	
4–6	45	56	
7–9	34	2	
≥10	9		

Supplementary Table 2. Clinical and histological characteristics of chronic hepatitis C patients before and after treatment (n=58)

**Characteristics**	**Baseline n = 58 patients**	**Week 24 n = 58 patients**	***P* value**
Age(yr)	41.5±14.1	–	-
Male, n(%)	31(53.5)	–	-
ALT, U/L	64.7±42.9	19.2±14.2	<0.001
AST, U/L	48.0±25.7	23.8±18.9	<0.001
Albumin, g/l	45.5±3.5	46.9±3.5	0.004
TBIL(μmol/L)	13.2±5.9	13.7±6.5	0.425
Platelets($\times 10^3$μL)	182.2±57.9	188.1±56.9	0.359
INR, ratio	1.0±0.1	1.1±0.1	0.003
HCV-RNA, Log IU/L	6.45±0.7	0±0	<0.001
Ishak fibrosis stage, n (%)			<0.001
0	1	4	
1	5	8	
2	4	5	
3	10	12	
4	18	12	
5	4	5	
6	16	12	
HAI score, n (%)			<0.001
0–3	9	37	
4–6	18	19	
7–9	17	1	
≥10	14	1	
Treatment, n (%)			
SOF+RBV	21	-	-
DCV+ASV	14	-	-
GZR+EBRr	7	-	-
TMC435	16	-	-

Supplementary Table 3. The significant parameters for the analysis of regressive patients between CHB and CHC. The parameters with p<0.05 were highlighted. Chi-square test was used for the analysis.

	All patients	Patients with baseline fibrosis stages F0/1/2	Patients with baseline fibrosis stages F3/4	Patients with baseline fibrosis stages F5/6
StrLengthPT	0.559	0.775	0.878	0.034
StrLengthPTAgg	0.421	0.523	0.380	0.034
StrWidthPTAgg	0.718	0.523	0.815	0.034
%Periportal	0.233	0.750	0.326	0.011
%PeriportalAgg	0.239	0.223	0.234	0.011
#LongStrPeriportal	0.304	0.894	0.380	0.024
#ThickStrPeriportal	0.379	0.894	0.502	0.024
StrAreaPeriportal	0.233	0.750	0.326	0.011
StrWidthPeriportal	0.181	0.894	0.326	0.011
#StrPeriportalAgg	0.233	0.797	0.502	0.011
#LongStrPeriportalAgg	0.392	0.223	0.275	0.024
#ThickStrPeriportalAgg	0.335	0.750	0.502	0.011
StrAreaPeriportalAgg	0.239	0.223	0.234	0.011
StrLengthPeriportalAgg	0.346	0.124	0.189	0.011
StrWidthPeriportalAgg	0.304	0.167	0.234	0.011
#LongStrZone2	0.235	0.914	0.048	0.801
#ShortStrPericentralAgg	0.273	0.238	0.029	0.122
%CV	0.093	0.790	0.010	0.818
%CVAgg	0.141	0.655	0.023	0.606
%CVDis	0.063	0.118	0.034	0.425
#StrCV	0.003	0.413	0.001	0.853

#ShortStrCV	0.014	0.797	0.010	0.606
#LongStrCV	0.013	0.750	0.001	0.920
#ThickStrCV	0.017	0.797	0.002	0.887
StrAreaCV	0.093	0.790	0.010	0.818
StrLengthCV	0.021	0.655	0.003	0.920
StrWidthCV	0.003	0.238	0.003	0.474
#StrCVAgg	0.005	0.391	0.002	0.920
#ShortStrCVAgg	0.078	0.517	0.036	0.639
#LongStrCVAgg	0.048	0.619	0.001	0.818
#ThickStrCVAgg	0.006	0.391	0.003	0.920
StrAreaCVAgg	0.141	0.655	0.023	0.606
StrLengthCVAgg	0.093	0.914	0.004	0.606
StrWidthCVAgg	0.029	0.529	0.014	0.697
#StrCVDis	0.015	0.311	0.027	0.818
#ShortStrCVDis	0.011	0.311	0.017	0.818
#LongStrCVDis	0.930	0.914	0.133	0.048
#ThinStrCVDis	0.049	0.397	0.036	0.639
#ThickStrCVDis	0.011	0.311	0.017	0.818
StrAreaCVDis	0.063	0.118	0.034	0.425
StrLengthCVDis	0.009	0.238	0.005	0.887
StrWidthCVDis	0.012	0.077	0.031	0.920
#IntersectionCV	0.042	0.653	0.017	0.920

Supplementary Figure 1. Flowchart of qFibrosis based on AI analysis of paired liver biopsy samples from patients with CHB and CHC

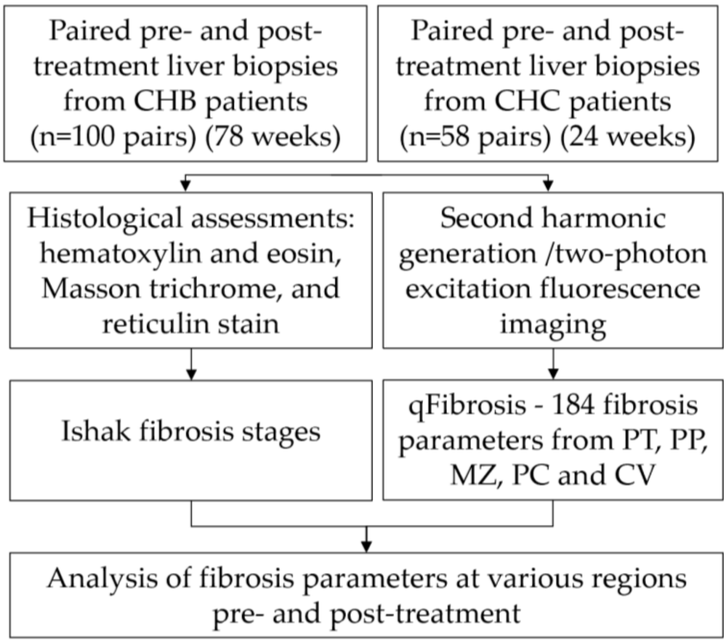

Supplementary Figure 2. Distribution of patients with fibrosis progression, stability and regression as determined by Ishak fibrosis score before and after treatment. CHB patients (A) and CHC patients (B). Fibrosis progression (yellow) or regression (green) was defined as increase or decrease in Ishak fibrosis score; fibrosis stability (brown) was defined as no change in Ishak fibrosis score compared with BL

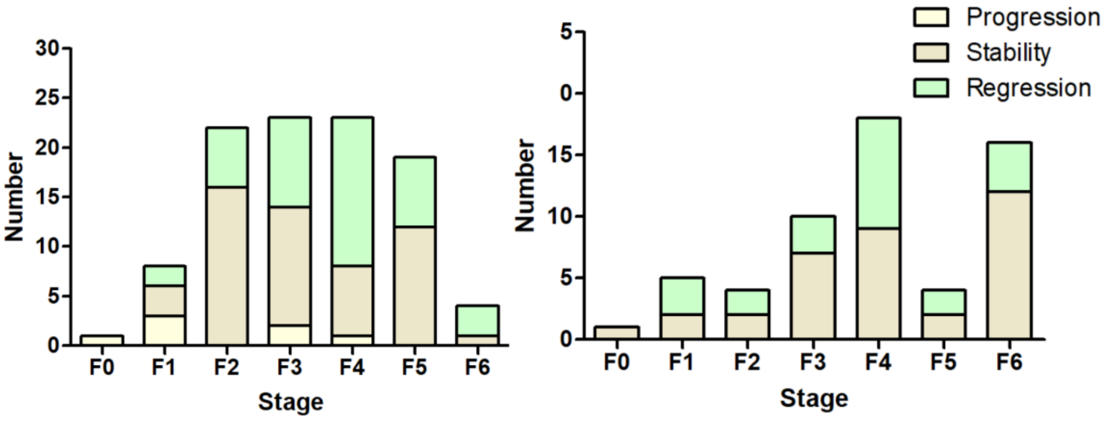

Supplementary Figure 3. Examples of pre- and post-treatment SHG/TPEF images from one CHB patient and one CHC patient. Both CHB and CHC patients had fibrosis improvement with 1-point Ishake stage and fibrosis regression at portal tract region (white arrow). CPA-PT, collagen proportionate area at portal tract region quantified by SHG/TPEF.

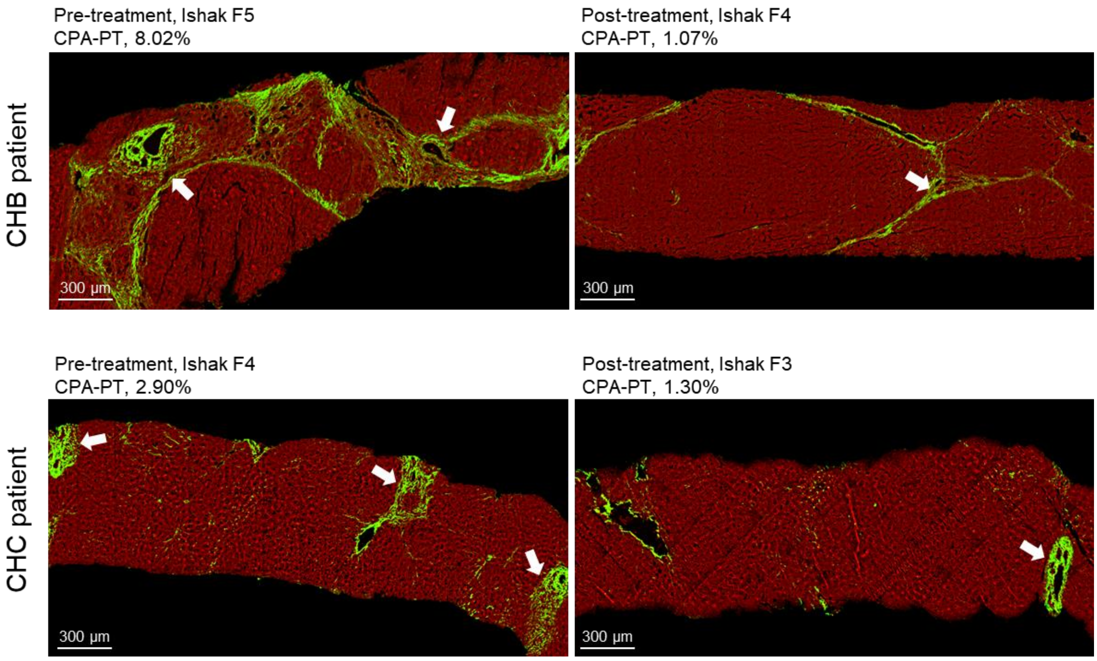

Supplementary Figure 4. Unpaired analysis - difference in collagen proportionate areas (CPA) and #string between treated and untreated liver biopsies from hepatitis B and C patients. **P*<0.05, ** *P* <0.01, *** *P* <0.001.

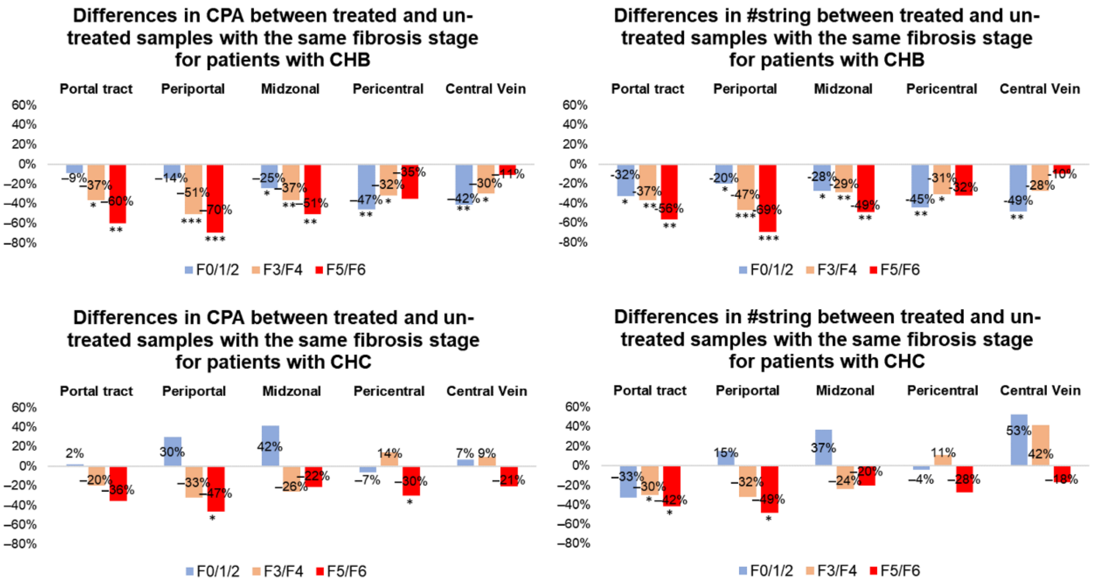

Supplement: Supplementary file 1 [file diagnostics-14-01837-s001.zip › diagnostics-3143685-supplementary.pdf]
